# Canine Distemper Virus in Autochtonous and Imported Dogs, Southern Italy (2014–2021)

**DOI:** 10.3390/ani12202852

**Published:** 2022-10-20

**Authors:** Flora Alfano, Gianvito Lanave, Maria Gabriella Lucibelli, Gianluca Miletti, Nicola D’Alessio, Amalia Gallo, Clementina Auriemma, Maria Grazia Amoroso, Maria Stella Lucente, Esterina De Carlo, Vito Martella, Nicola Decaro, Giovanna Fusco

**Affiliations:** 1 Dipartimento Coordinamento di Sanità Animale, Istituto Zooprofilattico Sperimentale del Mezzogiorno, 80055 Portici, NA, Italy; 2 Dipartimento di Medicina Veterinaria, Università degli Studi di Bari, 70121 Valenzano, BA, Italy

**Keywords:** canine distemper virus, dog, animal importation, CDV Arctic-lineage, CDV H gene

## Abstract

**Simple Summary:**

In the period 2014–2021, the circulation of CDV in dogs of Southern Italy was investigated. In this time span a reduction in the circulation of CDV was observed, with a higher frequency of detection of the pathogen in imported dogs (18.4%) compared to stray (7.4%) and household (3.9%) animals. These results underline the effectiveness of the prophylaxis strategy on autochthonous dogs as well as the importance of continuous surveillance of CDV, especially in imported dogs.

**Abstract:**

This study aims to investigate the presence of canine distemper virus (CDV) infection in 949 autochthonous or illegally imported dogs from Southern Italy, over a period of eight years (2014–2021). CDV RNA was detected in 6.8% (65/949) of the animals tested, with no detection of CDV in dogs sampled in 2020–2021. The frequency of CDV detection was higher in imported dogs (19/103, 18.3%) with respect to stray (27/365, 7.4%) and household dogs (19/481, 3.9%). On sequence and phylogenetic analyses of selected strains, the analyzed viruses belonged to the Arctic clade, which has already been reported in Italy and in Europe. The results of our study may suggest a reduction of CDV circulation in Southern Italy, while at the same time highlighting the need for strict controls on dog importation, in order to prevent the introduction of viruses from endemic countries.

## 1. Introduction

Canine distemper virus (CDV) is an enveloped, negative-sense, single-stranded RNA virus belonging to the family *Paramyxoviridae*, genus *Morbillivirus* [1]. It was first described by H. Carré in 1905 [2] and it can affect a very broad range of host species worldwide [3,4,5,6]. *Canidae* and *Mustelidae* families are the most affected, but CDV has also been detected in the *Felidae*, *Viverridae*, *Procyonidae*, and *Ursidae* families [7,8]. CDV infections are present in genetic diversity worldwide [9,10,11,12].

Its genome encodes for six structural proteins: a single envelope-associated matrix (M) protein, two membrane glycoproteins [hemagglutinin/attachment protein (H) and fusion protein (F)], two transcriptase-associated proteins [the phosphoprotein (P) and the large (L) polymerase protein] and the nucleo-capsid (N) protein, that encapsidates the viral RNA [13,14]. The heterogeneity within the H genes allows the molecular typing of CDV. H-glycoprotein mediates the binding of the virus to receptors on the host cell and is composed of a short N-terminal cytoplasmic tail followed by a transmembrane domain and a large C-terminal ectodomain [13,15]. Sequence variations may affect the virulence, host range, and neutralisation-epitopes of CDV, and play an essential role in cell tropism [16]. CDV infection can be prevented by an adequate host immune response against protein H [15]. The fusion of the cell membrane with the viral envelope is promoted by the F protein that also promotes membrane fusion between the host cells, with formation of syncytia [17].

Twelve genetic lineages have been evidenced by molecular studies (America-1 and -2, Arctic-like, Asia -1, -2, and -3, Europe Wildlife, Europe-1/South America-1, South America-2, South America-3, South Africa, Rockborn-like) [11,18]. The majority of CDV field strains cluster into six major genetic lineages, designated America-1 and -2, Asia-1 and -2, European and Arctic [19,20,21,22,23,24,25,26,27,28,29,30,31]. America-1 CDVs have not been detected over the last five decades and it is not known whether they are still circulating in the field. In Italy, the circulation of 3 distinct lineages have been reported: Europe (also called Europe-1/South America-1), Europe Wildlife, and Arctic-like lineages [18,28,29,32,33,34,35]. In animal hosts other than dogs, such as wolves, Arctic-like lineage CDV strains have also been detected [10,36,37,38] in badgers [33] and tigers [39]. Europe and Europe wildlife lineage CDV strains have been also described in foxes [28] and wolves [38], and in bears [40], respectively.

The CDV genetic lineages are variously distributed according to geographic patterns, although not to species of origin. Disease prevalence exhibits temporal fluctuations and increases during the cold season. In the environment, CDV is quickly inactivated. Transmission mainly occurs by direct animal-to-animal contact or by exposure to respiratory and ocular fluids and exudates, while other body excretions and secretions (e.g., urine and faeces) [7] could also contribute to viral shedding during the acute phase of infection. In domestic dogs, trans-placental infection has also been documented [41]. This highly contagious pathogen enters the new host and promptly starts replication [42], resulting in severe immunosuppression. Targets of infection are mainly mucous membranes and lymphoid tissues. This can result in either subclinical infection (50–70% of CDV infections) or severe, often fatal, systemic disease, mostly observed in immune-compromised animals [43]. 

The severity of clinical signs is influenced by strain virulence, environmental conditions, host age and immune status [11]. The infection may be prevented by passive or active immunization and is not age-restricted [44]. Young pups are protected by passive immunity and most adult dogs are protected by vaccine immunization. Age-related susceptibility to infection (3–6 months old pups are more susceptible than older dogs) correlates with the decline in maternally derived immunity. Individual variations among the various strains are responsible for the differences in virulence or tropism rather than properties inherent to a given CDV lineage [27,45].

This study aims to investigate the presence of CDV infections in dogs in Italy, over a period of eight years (2014–2021) in order to better understand the virus origin and distribution among animal populations. In order to elucidate the genetic divergences, selected CDV strains were sequenced and their sequences were compared with those available in the GenBank.

## 2. Materials and Methods

A total of 949 dogs and 6374 matrices were tested for canine distemper virus (CDV) over a period of 8 years (2014–2021) (Table 1) at the Istituto Zooprofilattico Sperimentale del Mezzogiorno (IZSM), statal public institution that operates within the National Health Service. The samples tested in this study in the framework within the diagnostic activity of IZSM. Out of the 949 tested dogs, 858 (90.4%) were dead and the analyzed samples (intestine, spleen, liver, lung, kidney, brain, heart) were collected at necropsy. A total of 91 (9.6%) animals were alive and the following matrices were analyzed: oropharyngeal swab, oculo-conjunctival swab, rectal swab or faeces. Three hundred and sixty five stray dogs (38.5%) and 481 (50.7%) household animals were collected from Calabria and Campania regions of Italy, whilst 103 (10.8%) dogs were imported from Eastern Europe (Hungary and Romania) (Table 2). The samples were subjected to extraction of nucleic acids by means of an automatic extractor (QIAsymphony, Qiagen, Hilden, Germany), using the “Virus/Pathogen” kit (Qiagen). Viral RNA was detected by a real time RT-PCR [46] and the positive samples were submitted to RT-PCR protocol for amplification of the hemagglutinin gene of CDV [47] and sequence characterization for determination of the CDV lineage. In addition, the extracts were also analyzed for the following canine viruses, to evaluate the presence of co-infections: coronavirus (CCoV) [48,49,50,51], adenoviruses (CAdVs) [52,53], herpesvirus (CaHV-1) [54], rotavirus (RVA) [55,56], parvovirus (CPV2) [57,58]. For CPV2, a PCR able to discriminate between field variants and the vaccine virus was used [59].

The PCR products underwent agarose gel electrophoresis at 50 V for 90 min. PCR amplicons were visualized on a Gel Doc™ EZ (Bio-Rad Laboratories SRL, Segrate, Italy), and subsequently subjected to purification. Direct Sanger sequencing was performed in both directions by Eurofins Genomics (Ebersberg, Germany).

The online tools Basic Local Alignment Search Tool (BLAST; http://www.ncbi.nlm.nih.gov, accessed on 28 June 2022) and FASTA (http://www.ebi.ac.uk/fasta33, accessed on 28 June 2022) were used, employing the default values to find homologous hits. Sequence editing was carried out by Geneious Prime version 2021.2 (Biomatters Ltd., Auckland, New Zealand). The sequences were aligned with other CDV strains recovered from the GenBank database by Multiple Alignment using Fast Fourier Transform (MAFFT) [60]. The correct substitution model for performing phylogenetic analyses and evaluation of selection pressure on coding sequences was obtained using “Find the best protein DNA/Protein Models” of MEGA X version 10.0.5 software [61]. Maximum-likelihood method, Tamura-Nei 4-parameter model, a discrete gamma distribution with six categories with 1000 replicates as statistical support were used. Bayesian inference and neighbor joining methods for the phylogeny were also explored, exhibiting similar topologies; maximum-likelihood tree was finally kept.

## 3. Results

Overall, out of the 949 dogs tested in a period spanning from 2014 to 2021, 6.8% (65/949) animals were positive for CDV by real time RT-PCR. CDV was not detected in 2020 and 2021 (Table 1). CDV was unevenly identified in the lung, intestine, brain, spleen, liver, heart and kidney of the positive animals. The frequency of CDV detection was higher in imported dogs (n = 19, 18.4%) than in stray (27, 7.4%) and household (n = 19, 3.9%) dogs (Table 2). The highest number of stray (nr = 22) and imported (nr = 10) CDV+ dogs was identified in 2014 whilst CDV was detected with the highest frequency in household dogs in 2015. Identification of CDV occurred in stray dogs in the 2014–2016 period, whilst in household dogs CDV was identified from 2014 to 2019 with the exception of 2017. CDV RNA was retrieved in imported dogs from 2014 to 2017 with the exception of 2017 (Table 3).

The 65 CDV positive dogs exhibited the following co-infections: 20 had a single CDV viral infection, 23 dual viral infections, 21 triple viral infections and 1 dog (78870/2016), had a quadruple viral infection (CDV + CPV2 + CCoV + CAdV). Among the 23 dogs with double infection, there were two co-infection patterns: 20 animals were co-infected by CDV + CPV2 and 3 dogs by CDV + CCoV. Among the 21 dogs with triple viral infections, there were two patterns: 17 dogs were co-infected by CDV + CPV2 + CCoV and 4 dogs by CDV + CPV2 + CAdV. CDV positive dogs imported from Eastern Europe were 18/65 (28%), with 3 being co-infections with the pantropic canine coronavirus pCCoV (acc. N. 103480/2014, 15910-3/2015 and 78870/2016) [62]. 

A total of 13 CDV-positive samples were selected based on the viral load as assessed by real time RT-PCR, with a threshold cycle (Ct) lower than 25. These samples were used for RT-PCR amplification of the complete hemagglutinin gene of CDV. A total of 8/13 samples could be amplified by RT-PCR yielding visible PCR products under gel visualization, while only 6 were suitable for direct sequencing based on DNA yield. One sequence was of poor quality. The sequenced CDV strains, ITA/2015/dog/24100, ITA/2015/dog/15952-1, ITA/2015/dog/15952-4, ITA/2015/dog/15952-5 and ITA/2015/dog/15952-6 were all characterized as Arctic lineage. For these dogs, the following information was assessed in detail: breed, age, origin, organs, co-infections, anatomopathological lesions (Table 4).

All the five animals from Table 3 were puppies except the animal #24100, was 12-months old.

Four CDV strains (ITA/2015/dog/15952-1, ITA/2015/dog/15952-4, ITA/2015/dog/15952-5 and ITA/2015/dog/15952-6) showed the highest nt identities (99.8–100%) to the Italian strain ITA/2015/dog/8387cucciolo (KX943323) whilst the CDV strain ITA/2015/dog/24100 displayed 99.7% identity to strain ITA/2013/dog/BA201 (KM115534).

**Figure 1:**
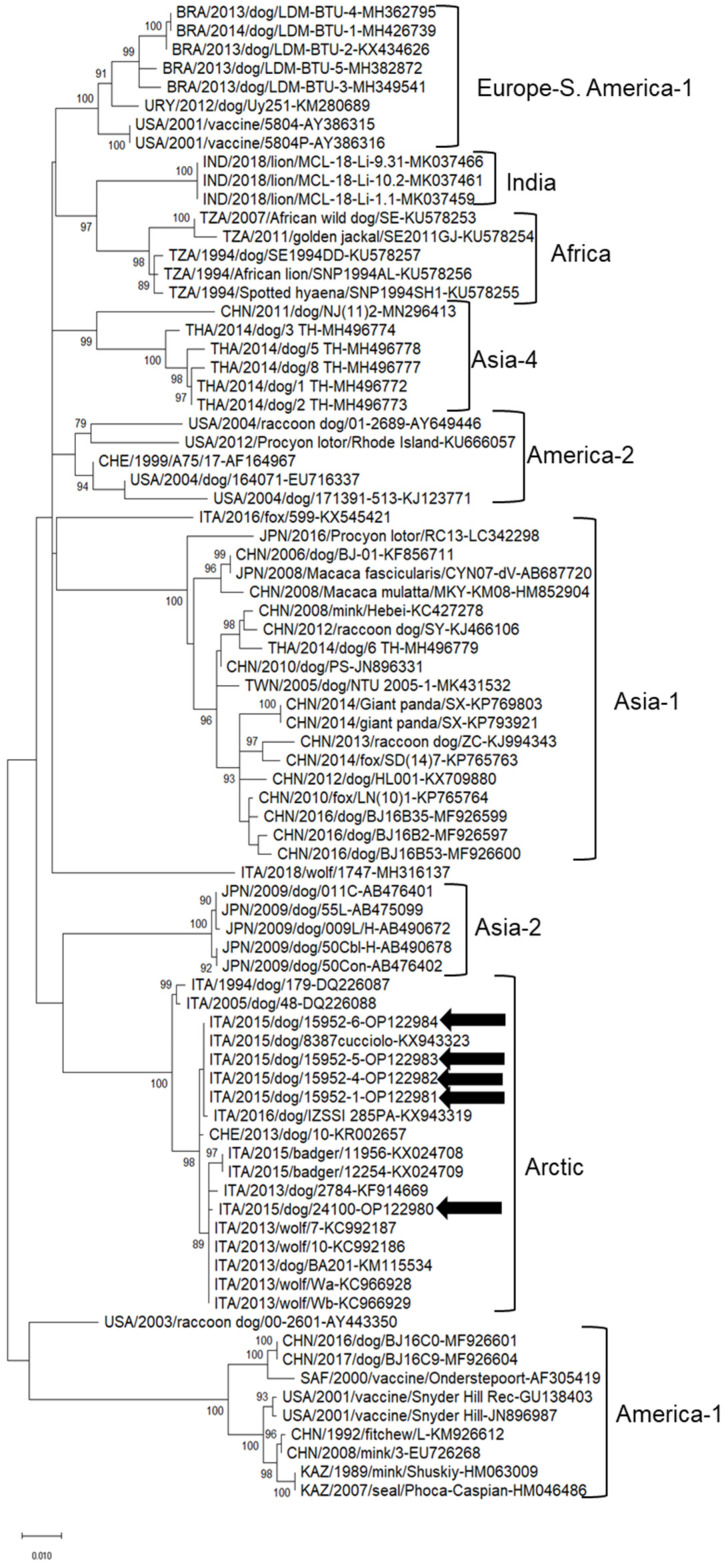
Phylogenetic tree based on the CDV partial nucleotide sequence of the hemagglutinin gene.

In the phylogenetic tree based on the partial nucleotide sequence of the hemagglutinin gene, the CDV strains identified in this study clustered into the Arctic clades together with strains retrieved in Italy from 2013 to 2016 and Switzerland in 2013 (Figure 1). The nucleotide sequences of strains ITA/2015/dog/24100, ITA/2015/dog/15952-1, ITA/2015/dog/15952-4, ITA/2015/dog/15952-5 and ITA/2015/dog/15952-6 were deposited in the GenBank database under accession nrs. OP122980-OP122984.

## 4. Discussion

The results of our study showed an active circulation of CDV in dogs in Southern Italy, either alone or in co-infection. Interestingly, we observed a marked reduction over the 8-year-long study period, with 37 cases in 2014, 20 in 2015, 5 in 2016, 1 in 2017, 1 in 2018 and 1 in 2019, with no cases in 2020 and 2021. CDV was identified in all the dog categories (household, stray, and imported animals) with peaks in 2014 and 2015 (Table 3). The higher frequency of detection of CDV in 2014 was mostly accounted for by infections in stray dogs (Table 3). A large distemper epidemic occurred in Italy during 2013 and involved primarily the Abruzzi region and neighboring regions, also affecting unvaccinated domestic and shepherd dogs, foxes (Vulpes vulpes), badgers (Meles meles), beech martens (Martes foina) and European polecats (Mustela putorius) [10,36]. The epidemic was sustained by an Arctic-lineage [34]. Therefore, it is possible that the peak of CDV activity observed in free-ranging dogs in Campania region in 2014 was somewhat linked to the epidemic observed in Central-Southern Italy in 2013. 

Alternate periods of quiescence and peaks of activity of CDV in the population are likely linked to changes in population immunity. Since vaccination against CDV is a core recommendation for dogs, most individuals are expected to be vaccinated and protected, and population immunity should be high enough to keep CDV infection under control, with only sporadic cases occurring [63]. When for some reason, specific population immunity decreases, the patterns of CDV infection may shift from sporadic to epidemic, posing a serious threat to susceptible populations [64].

Interestingly, out of the 65 CDV-positive dogs, only in 20 animals was CDV the only pathogen identified, with CPV2 and CCoV being frequently detetced in co-infection. Co-infections are frequently found in dogs [65,66] and the extent of this phenomenon has been revealed by the adoption of syndromic diagnostics (i.e., multi-screening of samples against a recognized panel of pathogens) [67] and, more recently, by metagenomic investigations in domestic and wildlife animals [68,69]. We cannot rule out that concomitant changes in the epidemiology of viruses with immune-suppressive activity may also occur in the local canine population, amplifying the magnitude of the CDV epidemic. For instance, both canine parvovirus and canine coronavirus are able to trigger leukopenia in dogs [13,70,71]. Functional imbalance of TCD4 lymphocytes after infection by the pantropic canine coronavirus can persist for 40 days [72], even if coronavirus infection is asymptomatic.

Co-infection in CDV-infected animals could be facilitated by animal life conditions/habits. Interestingly, the prevalence of CDV was higher in imported dogs (18.4%) than in free-ranging dogs (7.4%) and in household dogs (3.9%). Movements for animals can be very stressful and can decrease the immunological defenses [62]. Also, illegal trading of dogs is usually associated with low hygiene and health standards with incomplete or unknown vaccination status of young pups. By interrogation of metadata and animal history when available, we noted that in most imported dogs the vaccination schedule was incomplete or altered using false certificates.

Several lineages or genotypes of CDV exist that are variously distributed throughout several continents. The detection of Arctic CDV strains, formerly believed to circulate exclusively in the Arctic ecosystem, was first reported in dogs in 2004 and 2005 in an Italian study (29), and subsequently in the United States [31] and other European areas [73,74,75], raising questions on the origin of these unusual strains. Legal or uncontrolled trading of animals, as observed for pCCoV [62], may be linked to the observed changes in CDV epidemiology, as a consequence of the introduction of novel strains into CDV-naïve areas, or accounting for the resurgence of CDV in countries where CDV spread had been effectively and successfully controlled by vaccine prophylaxis.

We were able to determine the partial sequence of the H gene of a selection of CDV strains. All the sequences clustered within the Arctic-like lineage, along with CDV sequences obtained from other Italian studies. Interestingly, a unique strain, 24100 (accession OP122980) fell into a distinct sub-cluster, suggesting the circulation of different epidemic strains. 

## 5. Conclusions

Although vaccination against canine distemper has been used widely for many decades, this infection still represents an important disease for dogs. Several factors, including the high number of unvaccinated stray dogs present in Italy [76,77] and the illegal trading of dogs [62,78], may be related to the persistence and spread of CDV in the Italian canine population. Implementing controls on imported animals and enacting continuous surveillance plans, along with reinforced vaccination programs, would be necessary to defeat the threat of CDV. 

## Figures and Tables

**Table 1:** Dogs tested in the period 2014–2021, animals CDV+ and their relative frequency expressed as percentage.

Year	Dogs	CDV+ (%)
2014	186	37 (19.9)
2015	156	20 (12.8)
2016	111	5 (4.5)
2017	130	1 (0.8)
2018	88	1 (1.1)
2019	82	1 (1.2)
2020	99	0 (0)
2021	97	0 (0)
**Total**	**949**	**65 (6.8%)**

**Table 2:** Imported, stray and owned dogs analyzed by their geographical distribution and frequency distribution of CDV+ animals.

Category	nr of Dogs (%)	Geographical Distribution (nr)	CDV+ (%)
**Household**	481 (50.7%)	Campania, Italy (405)	19 (3.9)
		Calabria, Italy (76)	
**Stray**	365 (38.5%)	Campania, Italy (346)	27 (7.4)
		Calabria, Italy (19)	
**Imported**	103 (10.8%)	Eastern Europe (Hungary, Romania) (103)	19 (18.4)
**Total**	949 (100%)	Italy, Eastern Europe (Hungary and Romania) (949)	65 (6.8)

**Table 3:** Distribution over 2014–2021 of imported, stray and household CDV+ dogs.

Year	Category	CDV+ Dogs (%)
2014	Household	5 (13.5)
	Stray	22 (59.5)
	Imported	10 (27.0)
	Total	37 (100)
2015	Household	6 (30.0)
	Stray	6 (30.0)
	Imported	8 (40.0)
	Total	20 (100)
2016	Household	3 (60.0)
	Stray	2 (40.0)
	Imported	0
	Total	5 (100)
2017	Household	0 (0.0)
	Stray	0 (0.0)
	Imported	1 100)
	Total	1 (100)
2018	Household	1 (100)
	Stray	0 (0.0)
	Imported	0 (0.0)
	Total	1 (100)
2019	Household	1 (100)
	Stray	0 (0.0)
	Imported	0 (0.0)
	Total	1 (100)
2020	-	-
2021	-	-

**Table 4:** Information of dogs infected by CDV Arctic strain.

ID	Year	Species and Breed	Age	Origin	Organs CDV+	Viral Co-Infections	Non-Viral Co-Infections	Injury
ITA/2015/dog/24100	2015	DOG half-breed male	young adult (over 12 month)	Client-owner	Liver, lung, encephalus	absent	acanthosis nigricans, leishmania, tenia, hunting pellets, lead, neospora	encephalitis, pneumonia, enteritis
ITA/2015/dog/15952-1	2015	DOG akita female	puppy	Imported from Eastern Europe (Hungary)	lung, encephalus	CPV2a vaccinal and wild type	coccidia	enteritis, lymphoid atrophy of the spleen, bronchopneumonia, encephalitis
ITA/2015/dog/15952-4	2015	DOG chow chow female	puppy	Imported from Eastern Europe (Hungary)	lung, encephalus	CPV2a vaccinal	coccidia, klebsiella pneumoniae	enteritis, lymphoid hyperplasia of the milzam broncho-pneumonia, encephalitis
ITA/2015/dog/15952-5	2015	DOG pincher female	puppy	Imported from Eastern Europe (Hungary)	lung, encephalus	CPV2a vaccinal and wild type	coccidia	hemorrhagic enteritis with atrophy of the intestinal villi, lymphadenomegaly of the meseric lymphnodes, lymphoid hyperplasia of the spleen, broncho-pneumonia, encephalitis
ITA/2015/dog/15952-6	2015	DOG husky female	puppy	Imported from Eastern Europe (Hungary)	lung, encephalus	CPV2a wild type	coccidia	enteritis, lymphoid hyperplasia of the spleen, broncho-pneumonia, encephalitis

## Data Availability

The data presented in this study are available in this manuscript. Sequence data presented in this study are openly available in the GenBank database.
